# Medical Flossing and the Pilates Method: Their Effectiveness on the Strength, Endurance, and Functionality of Healthy Individuals

**DOI:** 10.7759/cureus.14758

**Published:** 2021-04-29

**Authors:** Lourdes Victoria Quiles-Sanchez, Ioannis Baroutas, Georgios Kyriakos, Nikolaos Gravvanis, Vasiliki E Georgakopoulou, Nikolaos Trakas, Christos Damaskos, Anna Garmpi, Nikolaos Garmpis, Vasileios Antoniou, Paraskevi Farmaki, Alexandros Patsouras, Errika Voutyritsa, Evangelos Diamantis

**Affiliations:** 1 Seccion de Endocrinologia, Centro de Salud Jesús Marín, Murcia, ESP; 2 Hellenic Police Academy, Athens, GRC; 3 Seccion de Endocrinologia y Nutrition, Hospital General Universitario Santa Lucia, Cartagena, ESP; 4 Department of Orthopaedics, Health Center of Peristeri, Athens, GRC; 5 Pulmonology Department, Laiko General Hospital, Athens, GRC; 6 1st Pulmonology Department, Sismanogleio Hospital, Athens, GRC; 7 Biochemistry Department, Sismanogleio Hospital, Athens, GRC; 8 Renal Transplantation Unit, Laiko General Hospital, Athens, GRC; 9 Department of Surgery, N.S. Christeas Laboratory of Experimental Surgery and Surgical Research, Medical School, National and Kapodistrian University of Athens, Athens, GRC; 10 First Department of Propedeutic Internal Medicine, Laiko General Hospital, Medical School, National and Kapodistrian University of Athens, Athens, GRC; 11 Breast Surgical Clinic, Saint Savvas Anti-Cancer Hospital, Athens, GRC; 12 First Department of Pediatrics, Agia Sofia Children’s Hospital, National and Kapodistrian University of Athens, Athens, GRC; 13 Second Department of Internal Medicine, Tzanio General Hospital of Piraeus, Athens, GRC; 14 Department of Endocrinology and Diabetes Center, G. Gennimatas General Hospital, Athens, GRC

**Keywords:** medical flossing, pilates, strength, functionality, endurance

## Abstract

The flossing method is an emerging therapeutic intervention based on the use of a floss-band that is circumnavigated at the various points of the body being treated. It is optimally combined with an appropriate exercise program to induce ischemia and release the fascia by applying pressure and movement to functional models. The Pilates method teaches the person to focus on the muscles, especially those responsible for the correct posture. It also helps the individual to become aware of the way he/she breathes. Both methods have positive effects on exercise and rehabilitation. However, medical flossing has not been as well researched as the Pilates method. This study aims to examine the effectiveness of both methods on the strength, endurance, and functionality of healthy individuals.

A review of the literature on medical flossing and Pilates was conducted. A systematic research took place from 2014 to 2019. Publications in non-English or non-Greek language were excluded. The articles were retrieved from not only PubMed, Scielo, and Elsevier databases, but also Google Scholar.

Both methods are understudied in relation to their effectiveness on the strength, endurance, and functionality of healthy individuals. More studies are required to estimate the effects of both methods on healthy individuals.

## Introduction and background

The flossing method is an emerging therapeutic intervention that started in the US and expanded in Europe by Medical Flossing, a training provider based in Germany in 2014. The method is based on the use of a floss-band that is circumnavigated at the various points of the body being treated. It is optimally combined with an appropriate exercise program and is integrated with a variety of manipulations and other therapeutic techniques to induce ischemia and release the fascia by applying pressure and movement to functional models [1].

The Pilates method, on the other hand, teaches the person to focus on the muscles, especially to control the muscles that are responsible for the correct posture and provide support to the spine. It also helps one become aware of the way he/she breathes and teaches him/her how to do it properly. The Pilates method has two types: “Fitness Pilates” and “Rehabilitation Pilates”. Fitness Pilates helps the healthy trainee get a well-trained body and also eliminate stress. “Rehabilitation Pilates” is a type of active rehabilitation for kinetic problems, usually after surgery or injury. The exercises of the Pilates method aim to improve the flexibility and strength of the body muscles without increasing muscle volume [2].

Both methods have been found to have positive effects on exercise and rehabilitation. However, medical flossing has not been as well researched as the Pilates method and especially on healthy individuals. Moreover, the Pilates method has been researched mainly for its overall effects and not specifically for strength, endurance, and functionality. This review aims to investigate the effectiveness of the flossing and Pilates method on the strength, endurance, and functionality of healthy individuals.

## Review

A systematic electronic research was conducted for articles from 2014 to 2019. Publications in non-English or non-Greek language were excluded. Reviews, case reports, clinical trials, and theses were considered eligible for the aim of this review.

The electronic databases utilized for this review were PubMed, Scielo, Elsevier, and Google Scholar. The keywords and terms used for the research were: “medical flossing”, “tissue flossing”, “pilates”, “healthy individuals”, “strength”, “endurance”, “functionality”. Duplicates were excluded. Moreover, articles including only abstracts or articles not being completely relevant to the management of medical flossing or Pilates methods were excluded. From the initial 378 articles found, only nine met the inclusion criteria.

Inclusion criteria

**Figure 1:**
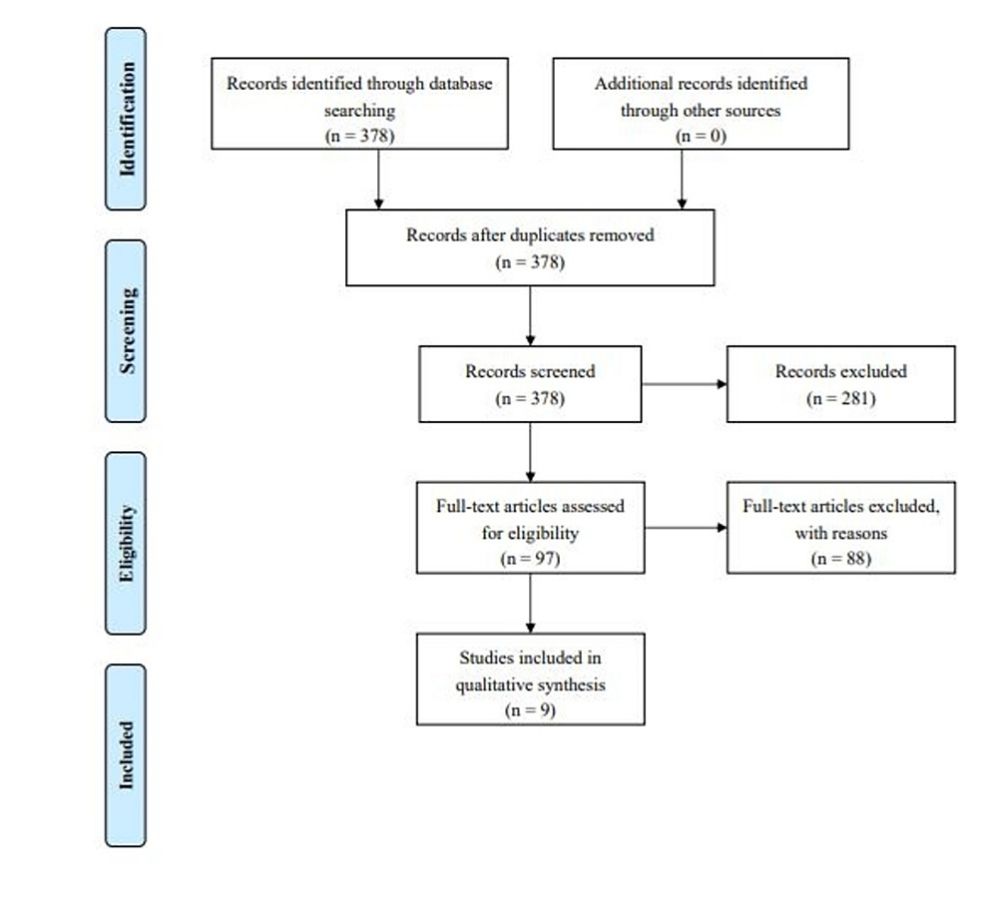
Prisma flow diagram for the current study. PRISMA, Preferred Reporting Items for Systematic Reviews and Meta-Analyses

The inclusion criterion was the use of floss bands or Pilates method during exercises that aim to enhance all or one of the following: strength, endurance, and functionality of healthy individuals. Studies on injured individuals were excluded. The process of finding and excluding articles can be seen on the PRISMA (Preferred Reporting Items for Systematic Reviews and Meta-Analyses) flow diagram (Figure 1).

Results

The research identified 378 articles, from which only nine met the inclusion criteria. Eight articles were about the medical flossing method and only one article considered the Pilates approach. Table 1 summarizes the results of the aforementioned studies.

**Table 1:** Studies on the outcomes of floss bands use and Pilates on healthy individuals. M, male; F, female; MFB, mobility floss bands; ROM, range of motion; IR, internal rotation; ER, external rotation; SD, standard deviation; RCT, randomized controlled trials; m, minute; WBLT, weight-bearing lunge test; CMJ, counter-movement jump; SPRINT, sprint test; FLOSS, application of floss band to both ankles; CON, without flossing of the ankle joints; SSHES, School of Sport, Health and Exercise Sciences

	Author	Year	Material	Method	Results
1	Bohlen et al. [3]	2014	5 participants (age: 20 ± 1 years; 1 M, 4 F)	14 days of once per day bilateral therapy that included: unloaded squats (2 sets x 10 reps), heel raises (1 set x 10 reps), active dorsi flexion (1 set x 10 reps), and passive ankle mobilization. During each session, floss bands were applied proximal and distal to the patella of the experimental leg.	Dorsiflexion peak torque increased 22% in the experimental leg (p = 0.06). The strength was modified.
2	Plocker et al. [4]	2015	17 M athletes	Participants attended a treatment session involving the use of MFB and a control session without the use of MFB.	The mean ROM was greater for both IR and ER for the treatment session (IR = 2.35 ± 11.33 SD; ER = 1.7 ± 8.29 SD).
3	Campos et al. [5]	2016	9 RCT	Review and meta-analysis	The results indicate the Pilates exercises performed on the mat or apparatus 2 to 3 times a week for 5 to 12 weeks improves abdominal muscular endurance (on average, 10 more abdominals curls in 1-m sit-up test) for both genders, when compared to no exercises.
4	Driller and Overmayer [6]	2017	52 recreational athletes	Participants performed a WBLT, plantar/dorsiflexion ROM and a single leg vertical jump test before and after the band was applied.	Significant improvement in all ROM measures as well as single leg jump performance after the use of a floss band.
5	Driller et al. [7]	2017	69 recreational athletes (32 M, 37 F)	Participants performed WBLT, CMJ, and a 15-meter SPRINT pre- and upto 45-m post-application of FLOSS or CON.	Significant intervention × time interaction in favor of FLOSS when compared to CON for the WBLT (p < 0.05).
6	Hodeaux [8]	2017	12 elite tennis players (6 M, 6 F)	The treatment trail had the floss band applied distal to proximal with approximately 50% tension on the band and each subsequent wrap overlapping the previous by ~50% covering the entire joint and securing the remainder of the band under the final wrap. Once the floss band was applied, the research assistant guided the participant through a series of six ROM exercises with three repetitions for each exercise with the band.	Some participants saw a mean increase in ROM after the use of the floss bands.
7	Ross and Kandassamy [9]	2017	10 participants (5 M, 5 F)	Participants had dorsiflexion ROM tested on either their right or left side (selection made at random) and then completed the intervention protocol on that side. All participants were then asked to return 72 hours later to have their opposite ankle assessed and to complete the intervention protocol. Immediately following pre-treatment ROM assessment, each client completed 150 seconds of Voodoo flossing.	The results showed that Voodoo flossing lead to a possibly moderate-sized beneficial effect, and most likely a small beneficial effect in the right leg and a most likely a moderate beneficial effect in the left leg. The outcomes of the research support the use of flossing for dorsiflexion increase.
8	Mills et al. [10]	2019	14 M professional rugby union athletes	Participants performed WBLT, CMJ, and a 20-meter SPRINT pre- and at 5- and 30-m post-application of FLOSS or CON on two separate occasions.	Benefits of floss bands when applied to the ankle joint to improve ROM, and jump and sprint performance in elite athletes for up to 30 m following their application
9	Stevenson et al. [11]	2019	5 athletes from Bangor University sports teams and the SSHES undergraduate programs.	Dorsiflexion, plantarflexion measurement with goniometer and WBLT with knee straight and bent.	The results showed that voodoo floss had a marginal to significant increase in the ROM of all ankle movements.

Discussion

The existing articles concerning the effects of floss bands are rather limited [3,4,6-9]. Bohlen et al. in 2014 studied the impact of two-week therapy of band flossing in combination with joint mobilization along with resistive workout on calf blood flow and plantar/dorsiflexion power. In this study, five participants were enrolled. Floss bands were practically used to the leg over the knee, whereas the subjects underwent a range of exercises, including unloaded squats, active dorsiflexion, heel raises, and passive ankle mobilization once every day. Then, the blood flow was estimated via the venous occlusion plethysmography procedure, and the strength was identified utilizing an isokinetic dynamometer. According to the results, the application of floss bands led to an increase in dorsiflexion peak torque up to 22%. Moreover, power in the leg over the knee was affected [3].

Plocker et al. evaluated the efficiency of tissue flossing procedure concerning not only the increasing upper extremity strength but also the range of motion (ROM). In this study, 17 male athletes participated. Athletes participated in two therapeutic procedures, one with the floss band and one without. During the therapy, participants’ shoulders were wrapped with the floss bands according to the guidelines. Then athletes underwent shoulder rehabilitation workout. After the floss band was removed, the ROM was estimated via a goniometer. Furthermore, upper extremity strength was also detected via a 3D accelerometer during a bench press. Plocker et al. demonstrated an increased internal and external shoulder ROM; however, a notable increase in ROM or upper extremity strength was not presented. It is worth mentioning that these outcomes are possibly affected by the fact that the recommended wrapping procedure did not successfully cover all the muscle moieties placed in the shoulder region [4].

Contrary to these findings, in 2017, Driller and Overmayer investigated the impact of floss bands on ankle ROM and jump height variation. In this study, 52 amusement athletes were enrolled. All the participants underwent a workout including a weight-bearing lunge exercise, plantar/dorsiflexion ROM and a single leg vertical jump exercise. These tests were performed before and after the floss band was used. Importantly, the floss band was performed to one ankle, whereas the other ankle was used as control. Athletes underwent 20 repetitions of plantar and dorsiflexion at the same time. Researchers noticed enhanced ROM measures. A single leg jump after the application of a floss band was also observed [6].

Driller et al. conducted a follow-up study in order to examine tissue ﬂossing presented on the ankle region ROM, and jump and sprint performance on 69 competitive athletes, including 32 males and 37 females. Athletes underwent a range of tests such as a weight-bearing lunge test (WBLT), as well as a counter-movement jump (CMJ) along with a 15-meter sprint test (SPRINT) pre- and up to 45 minutes post-performance of a floss band to both right and left ankles (FLOSS) or in the case that no flossing of the ankle joints (CON) took place. According to the results, there was an important intervention Β time interaction all for FLOSS in comparison with CON for the WBLT. These results were linked to insignificant or minor side effect at all time points. Minor but insignificant improvements were observed for FLOSS in comparison with CON for CMJ power (mean ± 90% CI: 89 ± 101 N) and 15-meter SPRINT times (0.06 ± 0.04 s) at 45 minutes post-performance [7].

Hodeaux examined the impact of floss bands on movement variation concerning the elbow region. Six female and six male elite tennis players participated. Measures of both elbow flexion and expansion, as well as pronation and supination in forearm ROM took place via a handheld manual goniometer. In order for the trustworthiness of the measures to be ensured, ROM measures were repeated three times and the average of the three measures was utilized. When baseline ROM was measured, participants were transferred to a different hall to undergo an intervention. After the floss band was performed, the tennis players underwent a range of six ROM exercises repeated three times. In some participants, a mean raise in ROM was observed after the floss bands were utilized [8].

Ross and Kandassamy examined the consequences of “Tack and Floss” active motion of joint on ankle dorsiflexion movement variation via Voodoo floss bands. In this study, 10 subjects participated, including five males and five females. Participants presented dorsiflexion, underwent ROM tests either on their right or their left side, and were randomly selected, and then the participants completed the intervention procedure on the selected side. Participants returned after three days in order for their opposite ankle to be tested. The time period of three days was chosen as sufficient washout duration in order for leftover side effects that may affect the untreated ankle to be avoided. Immediately after ROM treatment procedure performance every participant underwent 150 seconds of Voodoo flossing. According to the outcome, Voodoo flossing resulted in a potential mediocre improved effect, and most likely a small beneficial effect in the right ankle and a most likely a moderate beneficial effect in the left ankle. The results of the research support the use of flossing for dorsiflexion increase [9].

A year later, Mills et al. examined the impact of tissue flossing on ankle ROM, as well as on jump and sprint execution in elite union athletes of rugby. In this study, 14 male athletes were recruited and underwent a WBLT, as well as a CMJ test along with a 20-meter SPRINT. All the tests were performed pre-performance and at 5- and 30-minutes post-performance of a FLOSS or CON application on two districted cases. According to the results, no notable interactions were observed between the treatment method (FLOSS/CON) and time (5 minutes pre-performance and 30 minutes post-performance) for all the detected variables. Furthermore, the analysis of the size of side effects revealed minor improvements to FLOSS compared to CON for CMJ application 5 minutes post-performance (d = 0.28) and for 10 meters (d = -0.45) and 15 meters (d = -0.24) SPRINT time 30 minutes post-performance. Overall, the results were in favor of the FLOSS method in comparison to CON and CMJ [10].

Most recently, Stevenson et al. tested the application of Voodoo floss bands on the ankle’s ROM. Only five athletes from Bangor University sports teams and the School of Sport, Health and Exercise Sciences (SSHES) undergraduate programs took part in the study on the grounds that they had participated in vigorous activity including running, jumping, and weightlifting for at least three days. No significant differences were found between the floss (FLOSS) group and the control (CON) group prior to testing for all ankle movements. After the testing, a marginal or significant difference was found in favor of the FLOSS group for dorsiflexion, plantarflexion, straight leg WBLT, and bent leg WBLT. However, since the number of participants is too small and it is a convenience sample, there might be a selection bias and decreased external validity [11].

As far as the Pilates method is concerned, no recent original studies were found. The most original reference is a review article by Campos et al. For the comparison of Pilates versus control, favorable results were found in favor of Pilates for the outcomes: balance, muscle strength, trunk extensor and flexor muscles endurance, posterior trunk flexibility, hamstring flexibility, and quality of life. In the meta-analysis for the outcome abdominal muscular endurance, differences were found in favor of the Pilates method, with a difference of almost 10 abdominal sets in one minute. With respect to flexibility, results of the meta-analysis showed no differences between groups [5].

Medical flossing and the Pilates method have shown positive effects in the studies examined in this review. Medical flossing was mostly examined in relation to its effectiveness on ROM of joints of upper and lower extremities. The results have shown differences in ROM between exercises using floss-bands and regular exercises. However, none of the studies examined the benefits of the blood flow in specific regions and the response of hormones to floss bands.

The Pilates method, on the other hand, had more favorable results as far as a wide range of effects is concerned. It was found to have a positive effect on balance and muscle strength but not on flexibility.

## Conclusions

This review is the first to address the effectiveness of medical flossing and the Pilates method on the strength, endurance, and functionality of healthy individuals. The results show that there is a huge gap as far as research on this field is concerned. More studies are required to determine the impact of both medical flossing as well as the Pilates method on healthy individuals.
